# Pediatric Critical Care in Resource Limited Settings—Lessening the Gap Through Ongoing Collaboration, Advancement in Research and Technological Innovations

**DOI:** 10.3389/fped.2021.791255

**Published:** 2022-01-31

**Authors:** Ashley Bjorklund, Tina Slusher, Louise Tina Day, Mariya Mukhtar Yola, Clark Sleeth, Andrew Kiragu, Arianna Shirk, Kristina Krohn, Robert Opoka

**Affiliations:** ^1^Department of Pediatrics, Hennepin Healthcare, Minneapolis, MN, United States; ^2^Global Pediatric Program, Division of Pediatric Critical Care, Department of Pediatrics, University of Minnesota, Minneapolis, MN, United States; ^3^Maternal and Newborn Health Group, Department of Infectious Disease Epidemiology, London School Hygiene and Tropical Medicine, London, United Kingdom; ^4^Department of Pediatrics, National Hospital Abuja, Abuja, Nigeria; ^5^Department of Pediatrics, Tenwek Hospital, Bomet, Kenya; ^6^Childrens Hospital of Minnesota, Minneapolis, MN, United States; ^7^Department of Pediatrics, Africa Inland Church Kijabe Hospital, Kijabe, Kenya; ^8^Department of Internal Medicine, University of Minnesota, Minneapolis, MN, United States; ^9^Department of Pediatrics, Makerere University, Kampala, Uganda

**Keywords:** pediatric critical care, low and middle income countries, telemedicine, simulation, device innovation, virtual platforms, medical education, global health

## Abstract

Pediatric critical care has continued to advance since our last article, “*Pediatric Critical Care in Resource-Limited Settings—Overview and Lessons Learned”* was written just 3 years ago. In that article, we reviewed the history, current state, and gaps in level of care between low- and middle-income countries (LMICs) and high-income countries (HICs). In this article, we have highlighted recent advancements in pediatric critical care in LMICs in the areas of research, training and education, and technology. We acknowledge how the COVID-19 pandemic has contributed to increasing the speed of some developments. We discuss the advancements, some lessons learned, as well as the ongoing gaps that need to be addressed in the coming decade. Continued understanding of the importance of equitable sustainable partnerships in the bidirectional exchange of knowledge and collaboration in all advancement efforts (research, technology, etc.) remains essential to guide all of us to new frontiers in pediatric critical care.

## Introduction

Three years have passed since our article “*Pediatric Critical Care in Resource-Limited Settings—Overview and Lessons Learned”* was published (1). That article reviewed the history of pediatric critical care (PCC), discussed recent advances in PCC, as well as highlighting the expanding gap in the level of care available in pediatric intensive care units (PICUs) in high income countries (HICs) vs. low-and-middle income countries (LMICs). An overview of PCC in LMICs was discussed in regard to the current state of *staff* (properly trained and compensated medical staff), *stuff* (appropriate medical equipment), *space* (clean environment to treat patients), and *systems* (infrastructure and logistical organization to provide the services) with an emphasis on the lessons that have been learned and opportunities that remain in advancing PCC in resource-limited settings (1). Since its' publication, the field of PCC medicine has continued to advance rapidly through the use of quality improvement initiatives, advanced training programs with an emphasis on readiness and simulation, clinical research, and ongoing advanced technology development (i.e., non-invasive monitoring devices, extracorporeal support) and big data.

In this current update article, we discuss selected areas in which PCC has continued to advance in LMICs with aim of highlighting important advancements that have been made while emphasizing areas amendable to future growth. To focus the discussion and build on the prior review, we will highlight three areas of significant developments– research, training, and technology. The discussion will highlight lessons learned, as well as on the horizon innovations and opportunities for further engagement. Underscoring all these advances, and the momentum to propel them forward, has been a renewed understanding of the importance of equitable and sustainable partnerships in place of colonialism and paternalistic endeavors. It is important to point out that the authors' experiences are mixed (some based in primarily LMICs, others based primarily in HICs) thus this review is written from the mixed perspective of researchers/clinicians who have worked in LMIC-HIC partnerships over the past several decades.

Additionally, while the COVID-19 pandemic has created many challenges in healthcare, the alternative routes needed to accomplish research, training, and provision of care to larger populations in resource constricted situations has brought with it new innovations that have the potential to have a sustainable positive impact on PCC in these settings. In some ways the world became smaller with rapid transition to virtual meetings and trainings, creating a platform for rapid dissemination of knowledge. The possibility of HICs having a shortage of oxygen and lifesaving equipment, such as ventilators, sparked an interest in and support for bioengineers and medical device developers in HICs to focus energy inventing alternative low-cost options for costly technology. This is not mentioned to lessen the distressing impact of the pandemic, but rather to emphasize that with focused, motivated, supported effort, seemingly impossible hurdles can be overcome, and disparities lessened.

## Research

### Role of Research in Advancing PCC in LMICs

Clinical research has an essential role in the advancement of PCC. Understanding how our therapeutic choices impact clinical outcomes provides a framework for future study, guides therapies and informs protocol development. The importance of clinical research has been understood and supported financially by large institutions throughout HICs for decades, however the support for such research in LMICs has lagged. As noted in *Challenges and Priorities for Pediatric Critical Care Clinician-Researchers in Low and Middle Income Countries*, an article published in 2017 and written by authors from the Pediatric Acute Lung Injury and Sepsis Investigators (PALISI) research network (2), more data on both clinical care outcomes and treatment in LMICs is needed. Local research is essential in advancing critical care in LMICs, as outcomes from clinical research performed in HICs often do not translate directly to LMICs. The infrastructure, supplies and funding needed to conduct research in LMICs has often been prohibitive. In addition, the ethical concerns of research in vulnerable populations with variable availability of local institutional review boards has been another area requiring thoughtful discernment and attention to detail.

Despite these barriers, there have been considerable advances in research in LMICs over the last decade, notably an increase in: (1) Support for and engagement in research pertinent to PCC based therapies and outcomes specifically for children in LMICs; (2) Recognition of the importance of the research agenda being directed by LMIC clinician researchers; and (3) Support for research efforts directed by LMIC researchers in their own environment. These advances all have their unique but monumental impact on the expansion of pertinent research in these settings. It is beyond the scope of this article to review all the important LMIC led studies; however, we have chosen to discuss a few examples to highlight the advancing research agenda in LMICs and the variety of ways it impacts the delivery of PCC.

#### Clinical Research Advancement Impact

One notable example of the impact of research in guiding and advancing PCC in LMICs was the “FEAST” trial (3). This trial was conducted in Uganda, Kenya and Tanzania and enrolled over 3,000 children with severe infection and impaired perfusion (3). The researchers found increased mortality with the administration of fluid boluses in this pediatric population (3) in contrast to findings of smaller studies in pediatric patients with sepsis in HICs, which supported the use of rapid, aggressive fluid resuscitation—and were informing the surviving sepsis guidelines already being rapidly rolled out in many countries. While there is ongoing discussion regarding differences in the study population, methods, and reasons for differing outcomes, giving some pause to widespread adoption of a “fluid restrictive” protocol, the trial results clearly changed guidelines for fluid therapy in septic children in Africa where fluid use is now much more judicious. Moreover, these results influenced resuscitation and sepsis guidelines in HICs, including the widely accepted Pediatric Advanced Life Support (PALS) guidelines and other protocols, which now encourage a more cautious approach to fluids in septic shock (4, 5).

Research in LMICs affirming the benefit of interventions recommended for children in HICs has also advanced PCC. As an example, while the use of intraosseous needles in acute pediatric resuscitation is widely accepted in HICs, it remains important to evaluate this practice in LMIC settings to ensure similar outcomes. A study by El-Nawawy et al., in Egypt (6), demonstrated statistically significant shorter time to vascular access, and decreased mortality in pediatric patients with sepsis with an intraosseous line vs. intravenous catheter. This in-country research helps to validate this practice and increase widespread acceptance in all settings.

Yet another area of advancement in clinical research in LMICs is the investigation of therapies employed in LMICs that may not be commonly used in HIC settings. Without clinical research on these therapies, the standard of practicing evidence-based medicine is difficult to achieve. One area now being studied, is the use of “bubble” continuous positive airway pressure (bCPAP) in children beyond the neonatal period. BCPAP is a low-cost respiratory support being utilized worldwide for neonates with respiratory distress with proven efficacy (7, 8). However, because older children in HIC have access to ventilator derived continuous positive airway pressure (CPAP) or mechanical ventilation, bCPAP is not used and not studied in HICs. In LMICs, as pediatric appropriate ventilators are often not available, bCPAP has been utilized to fill a gap. A variety of well-designed studies have recently looked at outcomes of children supported with bCPAP in LMICs (9–11). These studies have yielded variable results, from improved survival to increased complications. This highlights (1) the advancements seen in research in LMICs with a technology that has been used for many years now being evaluated for efficacy and (2) the need for additional robust clinical trials to assist in determining which patients would benefit from this support in this specific setting.

Finally, surfactant has long been established as a lifesaving therapy in neonatal respiratory distress syndrome, traditionally requiring intubation and mechanical ventilation. Over the past decade, clinician-researchers have explored innovative ways of delivering surfactant in the preterm infant. This research has allowed for expanded use of surfactant with results demonstrating the efficacy of two alternative methods of delivery, using a nasogastric tube or laryngeal mask airway, when intubation and/or ventilation is not feasible (12, 13). For a costly therapy like surfactant, knowing that the alternative, feasible methods of delivery are efficacious is essential to good stewardship of resources in all settings.

#### Networking and Research Agenda

Over the past decade, increased networking and partnership between researchers in LMICs and HICs has had a positive impact on PCC research worldwide. Both strive to improve critical care through research for the most disadvantaged patients. The PALISI network is a United States based group known for their large and impactful PCC studies with over 150 high impact publications and 30 active research studies (14). PALISI has expanded to include a Global Health Subgroup designed to focus on the global burden of pediatric critical illness and support investigators dedicated to advancing the care of critically ill children throughout the world. Investigators from around the globe who seek to collaborate with communities caring for critically ill children limited by geography, resources, or social constraints, work together on projects with a research agenda guided by partner institutions in LMICs. Their multi-county survey of PICUs provided the most comprehensive overview and understanding of the currently available resources and infrastructure for PCC across the globe (15). This study highlights how having a large network can more rapidly accomplish the goals needed to improve care and advance research.

Another successful example of partnership and collaboration advancing critical care research in LMICs is the Networking for Improving Critical Care Systems and Training (NICST) in Sri Lanka. NICST has trained over 4,500 nurses and doctors in acute and critical care skills. Beyond the clinical training this “collaborative of clinicians, researchers, and educational experts linked through continuous audit is designed to improve patient outcomes” (16). NICST research focuses on improving quality and access of acute and critical care services by combining “clinical medicine with health informatics, epidemiological and social science, along with health systems and improvement science” (17, 18).

Other large collaborations which have had substantial impact include the Essential Emergency and Critical Care (EECC) Collaborators and the World Federation of Pediatric Intensive and Critical Care Societies (WFPICCS). The EECC is a group of critical care providers from around the globe that worked together to provide recommendations for essential emergency and critical care requirements for care across all ages in all locations as stated in their consensus statement article published in BMJ global health in 2021 (19). The WFPICCS is a leading organization advocating for care of critically ill children across the globe that combines national societies into an international network. In addition to the examples listed above some other prominent programs include societies of critical care in India, Cuba, Argentina, Iran, Egypt, and Brazil.

#### Research Training and Financial Support

The importance of training and funding on research advancement cannot be overstated—with the right resources, LMIC researchers are building sustainable research capacity to progress the field of PCC medicine. Training programs for researchers in LMICs have increased over the past decade. The Academic Competencies Series (ACES) course is one example of training that is now available. This program enrolls early-career researchers registered at universities in Ethiopia, Zimbabwe, Malawi, and South Africa in a year-long training course that equips the them with the necessary skills to become productive researchers (20). Similarly, the National Institutes of Health (NIH) and the Gates Foundation have provided training specifically geared to build capacity of researchers in LMICs.

Funding has historically been a barrier for researchers in LMICs. There has been some increase in funding mechanisms and grant opportunities for studies designed to PCC in LMICs and financially support the researchers and support staff needed to conduct these studies. Some examples include the grants offered through the Thrasher Research Fund, the Doris Duke Research Fellowship, Fogarty Funding (NIH), Mobile Health: Technology and Outcomes in Low- and Middle-Income Countries (NIH), U.S. Agency for International Development (USAID), and Gates Foundation.

### Research: Lessons Learned and Where We Go From Here

While we highlight the advancements in research, many lessons have been learned along the way. “Well intended” research projects have “gone wrong” by either (1) not ensuring the research agenda is on point with the research needed, or (2) pulling important scarce resources for research (nurses, supplies administrative time, equipment, etc.) that negatively impact overall function of the system. The balance of these priorities is essential to consider when planning a research agenda.

Our shared experience has demonstrated that research partnerships work best when built on a solid ethical framework, coupled with “mutual respect and benefit, trust, good communication, and clear partner roles and expectations” as described in *Successful Global Research Partnerships: What makes them work?* (21) The goals of any research conducted must first and foremost provide benefit to the population being studied and be driven predominately by the stated needs of the LMIC partner. Additionally, representation in the whole research cycle must be equitable including the opportunity for the LMIC partners to be principal investigators, first and senior authors, and present their work in national and international meetings. Depending on each researcher's starting point, whether from a LMIC or HIC, moving into key leadership positions in research requires training and time. Mentorship into these leadership roles should be the goal and outcome as soon as possible in these relationships, with the recognition that mentorship must be bidirectional.

Understanding challenges to all aspects of conducting research—such as implementing study protocols, consent, development of standard operating procedures, budgets, data collection and entry, and database maintenance is essential. This is key for all research, but especially to modify HIC practices to fit in resource constrained settings. Additionally, grant office support should include the researcher and the research, as well as administrative staff to avoid overstretching already limited resources. Partnerships ensure context-specific ethical issues are addressed for all settings.

Other vital aspects include engaging the regulatory bodies of all countries involved in the research. Some studies have demonstrated or highlighted challenges that can arise when ethical issues related to research in a vulnerable population and other local regulations are not adequately addressed prior to the study starting (22–24). Multinational studies may have different regulations for each country which can create challenges in satisfying all involved agencies [i.e., investigational review boards, clinical trials.gov, agencies regulating drugs and devices such as the Federal Drug Administration (FDA) or the equivalent LMIC agency; rules and regulations regarding importation of supplies and equipment necessary for the research].

As modeled by successful LMIC researchers such as Professor Philippa Musoke *(Makerere University, Kampala, Uganda), Executive Director for the Makerere University-Johns Hopkins University Research Collaboration (MU-JHU), Kampala and* Associate Professor Nadia Sam-Agudu *(University of Maryland School of Medicine, Baltimore, MD, USA), Senior Technical Advisor, Pediatric HIV, Institute of Human Virology, and International Senior Technical Advisor, Pediatric and Adolescent HIV, Institute of Human Virology Nigeria* both of whose publications span a wide range of research topics including but not limited to HIV and education (21, 25–28), providing equitable instruction/education for all team members in the details necessary to conduct successful and ethical research is essential. This must include fostering and role-modeling open regular communication with the entire team and following good research practice protocols including data integrity evaluation and study protocol adherence. Unforeseen circumstances may arise (power outages, supply shortage, broken equipment) and it is important to quickly recognize when a study protocol needs to be modified or additional training needs to be provided to ensure accurate data collection and results. These two exemplary researchers are among many others who have demonstrated lifelong achievement in research that should be looked to as a model for junior researchers.

Despite the numerous granting agencies willingness to fund LMIC-led projects, it remains difficult to find grants that are appropriate for funding some well-designed and pertinent studies. Developing resources and experts to guide the process of finding funding for important projects, writing grants that score well, and executing grants remain a challenge for both HIC and LMIC researchers.

## Training

### Role of Training in Advancing PCC in LMICs

Specialty specific training is essential for provision of quality PCC. While PCC physician training is important, multidisciplinary training is even more critical. Well-trained nurses form the backbone of every PICU in the world. Training can be delivered in multiple different ways but having training include nurses and providers together increases the learning for all, as trainees learn from each other's different perspectives. Education must be guided by the learner, with some learning best through case-based presentations, others with hands on and simulated experiences and still others learning best through a more formal curriculum which encompasses the use of lectures and independent reading (books, online, etc.). All these methods can be effective if they meet the learner where they are and build sequentially on their knowledge, experience, and current practices.

#### Training Standards and Guidelines

The recently published *Standards for improving the quality of care for children and young adolescents in health facilities* from the World Health Organization (WHO) creates a framework that PCC training programs can utilize to determine their educational goals. Training relevant to pediatric intensive care is captured by 24 quality measures (Table 1) (29), relating to eight quality standards and specify that the frequency of in-service refresher training is ideally at least once every 12 months. The breadth of recommended training covers the wide range of skills, knowledge and attitudes needed for multidisciplinary PCC health professionals to be competent, motivated, and empathetic (Standard 7). *Evidence-based practices (Standard 1) training relevant to PCC health workers is specified across a continuum of critical care: triage, assessment, emergency treatment, monitoring, supportive care, infection prevention, unnecessary interventions. Illness management training for conditions that may require pediatric intensive care are specified: Injuries, neglect, violence, surgical conditions and the common LMIC co-morbidity of malnutrition*. These standards provide an outline of goals and objectives for comprehensive PCC training.

**Table 1:** Pediatric critical care relevant training measures in the “Standards for improving the quality of care for children and young adolescents in health facilities” document (24).

**Standard**	**WHO SSNB standard**	**Quality statement**		**Quality measure**	**Type**
1	Evidence-based practices and management of illness	1.1	All children are triaged and promptly assessed for emergency and priority signs to determine whether they require resuscitation and receive appropriate care according to WHO guidelines.	Proportion of all professional health staff who care for children in a health facilitywho received training or refresher courses in emergency triage, assessment and treatment or pediatric emergency care during the past 12 months.	Process/output
		1.7	All children at risk for acute malnutrition and anemia are correctly assessed and classified and receive appropriate care according to WHO guidelines.	The professional staff at the health facility who care for children receive training and regular refresher sessions in assessment, identification, appropriate management and follow-up of children with acute malnutrition at least once every 12 months	Input
		1.11	All children are screened for evidence of maltreatment, including neglect and violence, and receive appropriate care.	The health facility staff receive training and refresher sessions on screening, preventing, protecting and managing children with evidence of maltreatment, including neglect and violence	Input
		1.12	All children with surgical conditions are screened for surgical emergencies and injuries and receive appropriate surgical care.	Health professionals receive in-service training and refresher sessions in appropriate care of child injuries, trauma and other common pediatric surgical conditions at least once every 12 months	Input
		1.13	All sick children, especially those who are most seriously ill, are adequately monitored, reassessed periodically and receive supportive care according to WHO guidelines.	Health professionals receive in-service training and regular refresher sessions on patient monitoring and supportive care at least once every 12 months	Input
		1.14	All children receive care with standard precautions to prevent health care-associated infections.	Health professionals who care for children receive training in standard infection prevention and control at least once every 12 months	Input
		1.15	All children are protected from unnecessary or harmful practices during their care	Health care staff in the facility receive in-service training and regular refresher sessions on harmful practices and unnecessary interventions at least once every 12 months	Input
2	Actionable Health Information Systems	2.1	Every child has a complete, accurate, standardized, up-to-date medical record, which is accessible throughout their care, on discharge and on follow-up.	The health facility staff receive training and refresher sessions at least once every 12 months on the use of standardized medical records, including birth and death registration, and classification of conditions and diseases in accordance with the ICD	Input
		2.3	Every health facility has a mechanism for collecting, analyzing and providing feedback on the services provided and the perception of children and their families of the care received.	Health facility staff (clinical and non-clinical) receive training or orientation in customer service and provision of child- and family-centered care at least once every 12 months.	Input
4	Effective Communication and Meaningful participation	4.1	All children and their carers are given information about the child's illness and care effectively, so that they understand and cope with the condition and the necessary treatment	Health care staff receive training and regular mentoring or refresher training at least every 12 months in fully explaining a condition to children and their carers, giving “bad news” and supporting children and parents in coping with the information given.	Input
		4.1	All children and their carers are given information about the child's illness and care effectively, so that they understand and cope with the condition and the necessary treatment	Proportion of health care staff, by cadre and social professionals who received proper continuous training in communication and counseling	Process/output
		4.3	All children and their carers are enabled to participate actively in the child's care, in decision-making, in exercising the right to informed consent and in making choices, in accordance with their evolving capacity.	Staff who care for children receive orientation or training in patient-centered care and legal and medical ethical principles of autonomy, informed consent, confidentiality and privacy at least once every 12 months	Input
5	Respect, Protection and fulfillment of children's rights	5.1	All children have the right to access health care services, with no discrimination of any kind	The health facility staff receive training and periodic refresher courses on nondiscrimination practices, promoting equity and cultural competence	Input
		5.3	All children and their carers are treated with respect and dignity, and their right to privacy and confidentiality is respected.	Health facility staff are trained in providing care with respect for dignity and for maintaining confidentiality during the care of children and have received refresher training at least once in the past 12 months	Input
		5.3	All children and their carers are treated with respect and dignity, and their right to privacy and confidentiality is respected.	Proportion of health facility health care providers who have attended training or received orientation in respecting and protecting the dignity of children and their carers.	Process/output
		5.4	All children are protected from any violation of their human rights, physical or mental violence, injury, abuse, neglect or any other form of maltreatment	The health facility staff receive training and orientation in identifying, assessing and providing care and support for victims of any form of maltreatment and on child protection procedures	Input
6	Educational, emotional and psychological support.	6.1	All children are allowed to be with their carers, and the role of carers is recognized and supported at all times during care, including rooming-in during the child's hospitalization	The health facility staff receive training and regular mentoring or refresher training in children's rights, including the right not be separated from their parents, and also in parents' rights and responsibilities	Input
		6.3	Every child is assessed routinely for pain or symptoms of distress and receives appropriate management according to WHO guidelines.	The health staff receive training and regular refresher courses in assessing, preventing and controlling children's pain at least once every 12 months	Process/output
		6.3	Every child is assessed routinely for pain or symptoms of distress and receives appropriate management according to WHO guidelines.	Proportion of staff who have received training or refresher training in children's pain management and palliative care within the past 12 months	Process/output
7	Competent, motivated, empathetic, multidisciplinary human resources	7.1	All children and their families have access at all times to sufficient health professionals and support staff for routine care and management of childhood illnesses.	Proportion of nurses who care for children admitted to the facility who have had pediatric training or in-service medical education in child care	Process/output
		7.2	Health professionals and support staff have the appropriate skills to fulfill the health, psychological, developmental, communication and cultural needs of children.	Health professionals and staff who care for children in the health facility receive in-service training and supportive supervision with regard to the legal entitlements and rights of children in relation to health care.	Input
		7.2	Health professionals and support staff have the appropriate skills to fulfill the health, psychological, developmental, communication and cultural needs of children.	Proportion of health professionals who care for children who received in-service training and/or refresher sessions within the past 12 months	Process/output
		7.3	Every health facility has managerial leadership that collectively develops, implements and monitors appropriate policies and legal entitlements that foster an environment for continuous quality improvement	Proportion of staff members who gave positive feedback about internal policies and activities for continuous quality improvement, including on-the-job training and personal mentoring	Outcome
8	Essential physical resources for SSNB available	8.2	Child-friendly water, sanitation, hand hygiene and waste disposal facilities are easily accessible, functional, reliable, safe and sufficient to meet the needs of children, their carers and staff.	Proportion of health facility health professionals and support staff who received training or mentoring in sanitation, hand hygiene and infection prevention and control in the past 6 months.	Process/output
		8.4	Adequate stocks of child-friendly medicines and medical supplies are available for the routine care and management of acute and chronic childhood illnesses and conditions	Proportion of health professionals who provide child health services who have received training in appropriate child medication.	Process/output

#### Simulation Based Medical Training

Healthcare simulation is an area of fast growth in HICs with much advancement in fidelity over the course of the last decade. Simulation based medical education has allowed all levels of learners to acquire skills needed to perform high-risk procedures and has improved teamwork and comfort in high-risk, low volume clinical situations. Simulation technology and educational strategies are being increasingly adapted to low-resource settings (30). Simulation can be “hands on” (31), virtual reality (32, 33), or screen based (34). Organizations producing mannequins “not-for-profit” have helped advance “hands-on” simulation in LMICs in the last 10 years, as have trainings designed specifically for PCC in low resources settings (35).

The mannequin-based simulation training course, Helping Babies Breathe (HBB), which utilizes a low-fidelity, low-cost mannequin and equipment is a prime example of a high yield training which has assisted in training multidisciplinary healthcare workers in neonatal resuscitation techniques and priorities for LMICs. This training reinforces important concepts and has the essential fidelity. Utilized supplies allow for participants to demonstrate bag-valve-mask use with appropriate mouth/nose seal, feel a pulse on the umbilical cord, or hear the apical heart beat—the essential skills that the course wants to teach (36). Taking this one step further, this low-cost training equipment (including a bag-mask and penguin suction) is high-quality, re-usable equipment designed for actual patient care.

Standardized emergency and pediatric critical care curricula that can be adapted for use in LMIC settings provide comprehensive training. These trainings are often designed for the “non-intensivist” and are readily deployable and consistent. Successful examples include the Pediatric Basic Assessment and Support Intensive Care (BASIC) course, Pediatric Fundamentals of Critical Care Study (PFCCS) and Emergency Triage, Assessment, and Treatment plus (ETAT) course. BASICS was created by pediatric intensive care leaders from WFPICCS and has courses geared toward nurses and non-intensive care physicians. BASICS has shown success in building local critical care capacity through a “train the trainer” model in high, middle and low income countries. PFCCS was developed by pediatric critical care leaders from the Society of Critical Care Medicine and can be administered through traditional live, in person course or online. The course has been successfully run internationally by 30 institutions; however, the course must be administered by trained, certified instructors (rather than train the trainer) and the cost can be prohibitive (37). ETAT is a comprehensive training for healthcare workers practicing pediatric emergency care in low resource settings that is available for free online or with in person courses (offering hands-on or screen based simulation options). Recently the “Pediatric ETAT guidelines” have been updated (38). The materials are based on the WHO pediatric care guidelines and the training has been successfully implemented in many eastern African countries and is expanding (39).

Finally, other low-tech examples of teaching procedural skills, in addition to facilitating low-cost adaptations for commonly needed pediatric procedures, is provided for free, on the open-access web-based *Simulation Use for Global Away Rotations* (SUGAR) platform. Short videos are available under the *Procedural Education for Adaptation to Resource-Limited Settings* (PEARLS) tab, which review procedures and how they can be practiced for use in resource limited settings (40). These procedures and scenarios can then be practiced with mannequins and low-cost “task trainers”.

Improved internet and smart phone availability in many LMIC settings, that was unimaginable a decade ago, has made it possible to utilize relevant open-access distance learning training courses already developed for the PCC setting. The COVID-19 pandemic has illustrated the importance of the ability to rapidly update materials for emerging situations. An example of this being the rapidly developed, and quickly distributed, WHO Severe Acute Respiratory Infection Training materials (41, 42). Mobile phones apps of contextualized guidelines increase access to information when needed for decision support (43, 44). Innovative refresher low-dose high frequency training using mobile virtual reality simulation has been successfully piloted in LMIC for neonatal resuscitation including the recently released Essential Newborn Care Now (45, 46).

#### Specialty Training Programs

Formal specialty training programs such as PCC fellowships for physicians and dedicated pathways to train nurses and other healthcare providers have been limited in resource constrained settings. In a recent survey, by the PALISI Global Health Subgroup, Muttalib et al., reported that less than half of the 238 hospitals representing 60 countries had subspecialty acute care training (PCC or pediatric emergency medicine) (15). Pediatric acute care fellowship training designed for physicians in LMICs are increasing in number, though not nearly fast enough to meet the needs of populations they serve.

There are different models that can be utilized for establishing subspecialty training in LMIC. South Africa, Egypt and India, have developed programs in an LMIC context, therefore modeling new programs based on their model is one way forward. For example, Professor Andrew Argent and his team runs the “African Pediatric Fellowship Program” (47) out of the University of Cape Town with training based at the Red Cross War Memorial Children's Hospital. The fellowship offers different training options including a 2-year master's program or a 1 year post graduate diploma—both aimed at allowing practicing physicians to develop skills specific to management and advancement of care for critically ill children. A program in India similarly offers a 1 or 2 year clinically focused training option, in addition to more formal 3 year training (48). Additionally, there are increasing established nurse led academic initiatives with post graduate nursing diplomas in child nursing, critical care nursing, and a Master of Nursing in child nursing—again all aimed at decreasing under 5 mortality through improved critical care for children (49). They have established programs from this model in Kenya, Malawi, Nigeria, and Zambia as well. Additionally, in many LMICs, critical care is provided by anesthesiologists. Opportunities for combined pediatric critical care and anesthesiology training should be considered.

Finally, another notable example of a training model is the Pediatric Emergency and Critical Care-Kenya (PECC-Kenya) Fellowship training program. This two-year fellowship in Pediatric Emergency and Critical Care is customized for pediatricians from Sub-Saharan Africa, with training occurring at University of Nairobi/Kenyatta National Hospital and at A.I.C. Kijabe Hospital in rural Kenya. This fellowship uses the model of a collaborative effort between established national universities and a partner institution in the USA, bringing together national and international faculty to train the fellows. Without fellowship training programs such as this, physicians pursuing formal specialty training in PCC would often travel to HICs to train. Removing physicians for training out of country, burdens countries already struggling with critically low numbers of healthcare providers and even lower numbers of critical care providers—a concept referred to as “brain-drain.” Collaborative training programs that are less formal than PECC-Kenya, such as the Haitian Pediatric Critical Care Collaborative, with training occurring at Saint Damien's Pediatric Hospital on the outskirts of Port-au-Prince, Haiti, utilize a similar model of national and international faculty training local physicians to specialize in PCC through a combination of virtual and local training. The establishment of formalized fellowship programs, such as local programs described in Egypt, South Africa, and India as well as collaborative programs such as PECC-Kenya are advancing training in the field of pediatric critical care in LMICs in a sustainable manner.

As we previously highlighted in the last article, without trained PCC nurses it's impossible to have PCC. Much of that training has been on-the-job training but there are increasing efforts to provide more formalized training for nurses and other allied health professional in PCC. One of the stated objectives of WFPICCS is to “promote multidisciplinary collaboration between Pediatric Intensive and Critical Care specialists by supporting education and professional developments of all health professional involved in Pediatric Intensive (and Critical) Care” which they accomplish through lectures focused on nursing (38). One example of a short course specifically focused on nursing sick children is a 16 week course being offered through the University of Cape Town in South Africa (50). The school of Post Basic Nursing at the University Of Abuja Teaching Hospital also offers a 1 year program on Intensive Care Nursing. Longer more in depth courses are needed but these offerings are a start. Encouraging educational opportunities which include not only physicians but also nurses, and allied health professionals, is essential if we are to continue to advance care globally.

### Training: Lessons Learned and Where We Go From Here

While there are a variety of available PCC trainings, the importance of contextualization is essential for the training to be effective (51). “Off the shelf” short courses developed in HIC contexts will result in little sustained behavior change if local adaptation is overlooked (52). When collaborations with HICs are established for LMIC training, a participatory needs assessment is an effective way to invite mutual identification of learning needs, epidemiology, work environments, equipment, infrastructure and team composition (53). Inter-professional team-based learning, is most likely to overcome the systems barriers to transfer into the workplace (52). Partnering for curricula adaptation will enable training faculty and maximize sustainability (52). An excellent example of a partnership encompassing international HIC guidelines with local adaptations is the ETAT course which has resulted in improved outcomes in LMICs (54). There remains the challenge of measuring the impact of these trainings in regard to sustained changes in care patterns and improved outcomes in LMIC (52, 55).

Typically, PCCs short courses focus more on skills/doing (psychomotor) and knowledge/thinking (cognitive) domains of learning with less emphasis on the affective domain of attitudes/values. Including even short learning activities that connect to learners' values/attitudes can be a powerful learning experience e.g., “Start with a story” at the opening of the neonatal Helping Babies Breathe course (35). Normalizing these activities is also important to improve the support for health providers with emotional fatigue and burnout before they leave the PCC workforce.

Reviewing collaborative training partnerships with a decolonizing lens is vital. Long-term relationships, cultural humility and shared planning with genuine listening to expert PCC colleagues in LMIC is only the beginning of that journey (56). Barriers to international travel for PCC training opportunities disproportionately affected health care providers from LMIC including funding and visa regulations (57). During the COVID-19 pandemic, inequitable vaccine availability, as well as nation-specific travel regulations, have further hampered calls for bidirectional training opportunities and affected training collaboration exactly at the time health workers in both HIC and LMIC settings need it the most.

## Virtual Technology, Collaboration and Resource Sharing

### Role of Virtual Technology, Collaboration, and Resource Sharing in Advancing PCC in LMICs

It has long been realized that sharing information and knowledge is essential to advancement of PCC. Over the last decade, the advancement in technology has made the ability to collaborate and share resources much easier. Worldwide there has been significant improvement in internet access and platforms have been created to easily interact across the globe.

#### Telemedicine

A major challenge in the provision of critical services has been the lack of adequate numbers of intensive care physicians. This is particularly concerning in LMICs. In HIC's the absence of intensive care coverage in remote areas where an intensivist is absent has been managed in part through the employment of telemedicine and other virtual platforms (58–60). Telemedicine is essentially the delivery of care to critically ill patients by critical care providers located remotely to fill gaps in healthcare services (58–61). This important critical care innovation offers an opportunity for the provision of critical care services even in the most remote parts of our world.

ICU telemedicine providers employ the use of electronic medical records (EMRs) in combination with audiovisual tools to assist caregivers at the bedside with patient care, clinical monitoring, and the development and implementation of care plans (60). Since its rollout, the growth of telemedicine as a modality of care delivery has evolved and encompasses care across multiple electronic platforms and clinical settings (59, 60). A variety of models of telemedicine have been in use from a centralized, single remote center providing care to multiple other locations (59, 60), to a remotely located intensivist virtually reviewing individual patients using an audio-visual connection either via computer or mobile device (59, 60).

Cellular and satellite technology has been used to assist in the provision of critical care in disaster settings (59). An example of the successful use of Tele-Pediatric Intensive care came from its' use in war torn Syria where a volunteer core of physicians provided consultation to medical directors in field hospitals regarding multiple management decisions such as mechanical ventilation settings, vasoactive medications, resuscitation, and intravenous fluid management (62). When high-definition video failed due to limited internet connectivity they quickly resorted to text, voice, and photo-based interactions such Facebook^TM^ messenger and Skype^TM^ to facilitate communication (63).

Unfortunately, traditional telemedicine services can be cost prohibitive for remote needs in both HIC and LMICs, which has presented widespread implementation (59–61, 64). Low-cost innovations for telemedicine are on the horizon. One example, utilized in Kenya, is a mobile application (app) called “Daktari Popote” (Doctor Anywhere)- which gives users access to medical specialists for a consultation. The app allows users to upload photos and x-rays, as well as receive prescriptions. Development of these app based virtual care platforms enable patients increased access to care (65). Similar applications and telemedicine consultative services have been developed and utilized by Medicin Sans Frontieres and Enlace Hispanico Americano de Salud (Hispano American Health Link) (EHAS), as well as other international collaborative groups. Best practices for use of these applications are still being developed (66, 67).

The covid pandemic has revolutionized telemedicine on a global scale. It has also been an opportunity for the promotion and validation of telemedicine technologies (61, 68). By necessity, clinical care had to adapt to the pandemic-related shutdowns of clinics and limitations of access to hospital services. Globally, healthcare systems have had to adapt to this new paradigm. The employment of platforms such as Zoom^TM^, Cisco WebEx^TM^, Microsoft Teams, Google Meet^TM^, GoToMeeting^TM^ and apps such as WhatsApp^TM^ and FaceTime^TM^ among others have enabled critical care professionals to fairly seamlessly share information and collaborate in patient care. Caveats remain, as these easy-to-use platforms are not designed specifically for sharing personal health information and they need to be equipped with a manner to protect patient information. There is password protected and Health Insurance Portability and Accountability Act (HIPAA) compliant versions that should be utilized as feasible and attention needs to be paid to where information is being broadcast or discussed (just as with in person care). Of course, having an appropriately trained PCC physician at the bedside is still the best practice care plan, however, the ability to reach areas with no access to such a resource is a huge advancement for PCC during times of resource constraint or in areas that have limited care provider availability.

#### Virtual Conferences

While virtual platforms for clinical care have advanced rapidly, the virtual education opportunities are even more expansive. Online learning opportunities have been expanding over the last decade, but with the pandemic even more virtual options have been created. The ability to participate in virtual conferences has meant that major impediments to clinicians from LMICs participating in international medical society meetings, such as the cost associated with travel abroad, the ability to leave heavy patient care responsibilities, and travel restrictions, have been eliminated. Time zone constraints can be overcome through creating recorded “on-demand” resources. Costs of virtual attendance, however, need to be adjusted to enable those in LMICs to attend.

One example of how virtual platforms can quickly impact knowledge dissemination occurred during the early days of the pandemic. One of the authors (AK) participated in a number of weekly Zoom calls set up by a tertiary care center in Kenya to share information with clinicians around the country about COVID. These Zoom sessions featured speakers from Kenya as well as other countries in Africa, China, the US, and Europe. They were attended by hundreds of clinicians across Kenya, from remote district hospitals to larger referral centers, and had a significant impact in preparing Kenyan clinicians for managing patients and protecting themselves from infection.

Similar collaborations between American Academy of Pediatrics (AAP) and The Pediatric Association of Nigeria (PAN) via the ECHO platform (a telemedicine/teleconferencing like technology) facilitated virtual learning to hundreds of healthcare workers on the topic of COVID-19 in children, with speakers and case presentations from Nigeria and various parts of the world (69). Additionally, the International COVID-19 PICU Collaborative, a voluntary collaboration was formed with hundreds of pediatric critical care medicine and infectious disease providers from around the world to share best practices and real time information across the globe. This group met via virtual platform on a weekly basis to rapidly share best practices and real-time information while sharing characteristics of the pediatric patients they were seeing in the early pandemic. The rapid formation of these global collaborations demonstrate how virtual platforms can bring educational resources rapidly together in real-time.

#### Online Learning—Sharing Knowledge and Resources

Over the past decade, opportunities to share PCC educational materials and global health educational materials online have increased dramatically, again with rapid advancement during the COVID-19 pandemic. Some schools have created entire online PICU curricula (70), while others have utilized online education to augment PICU residents' understanding of certain topics (e.g., mechanical ventilation) (71). Others created online global health educational resources designed to be widely shared and used in various settings. Courses ranged from pre-travel education (and simulation as discussed in training above) to prepare American residents for working abroad to free PICU critical care cases with open access, and online continuing medical education for critical care nurses (72–74). During the COVID-19 pandemic many medical education centers moved to online education for a myriad of topics, including video conferences (75), joint online training and projects between global health partners in more than one physical location (76).

An excellent example of an established online resource for sharing clinical education is OPENPediatrics (www.openpediatrics.org) which was developed by Boston Children's Hospital (77). This site offers access to peer-reviewed content, including accredited and non-accredited courses, expert lectures and demonstrations, interactive device simulators, protocols and medical calculators. Additionally, the Twitter hashtag for Free Open Access Medical Education (78) (#FOAMed) has been widely used to rapidly disseminate medical information to medical practitioners around the world, particularly emergency medicine physicians, including to physicians in low resourced settings (78) however, this remains controversial due to concerns about maintaining quality (79). MedTwitter, in particular #PedsICU, has been used to rapidly share PICU relevant information around the world during the COVID-19 Pandemic (80).

### Virtual Technology Online Education, and Educational Collaboration and Resource Sharing: Lessons Learned and Where We Go From Here

Technology in many ways has made the world a much smaller place. Collaboration in patient management and in medical training and education can literally happen in real time across the globe and across multiple time zones. Challenges remain in the use of telemedicine including developing ways to protect patient information, increasing the availability of computer and mobile technology in LMICs, and ensuring adequate internet and cellular service connectivity.

While access to online educational material is an opportunity to expand available information, which aspects of pediatric intensive care can be taught well-online remains to be elucidated. Studies of medical trainees' knowledge, attitudes, and practices comparing in-person to online training are currently being done (81), and will continue to be studied for years to come. Once again, the applicability of the material to the setting in which one is practicing or the resources available in that setting make some topics less ideal for “non-individualized” virtual/online education. Continued development of these resources by clinicians in LMICs is essential to broadening the applicability of this method of education.

## On the Horizon Developments

As excited as we are about the advancements that have taken place over the past decade, there are other “on the horizon” developments that have the potential to significantly advance PCC in LMICs even further over the coming decade. As we conclude this article, we have highlighted a few of the advancements that we believe will have a large impact in the coming years.

### Electronic Medical Records

With the advancement in telemedicine and virtual training/education platforms, a push for development of electronic medical records becomes more salient. EMR is a technology that becomes increasingly important in critical care as the share of multidisciplinary, medically complex, and chronic conditions increase. In addition to the commonly recognized benefits, such as the ability to track labs, images, and other information across time and improved legibility, other benefits include the facilitation of telemedicine (virtual private networks), tele-radiology, and research and quality improvement initiatives.

However, like any other infrastructure, EMRs come at a cost. There is a cost to initial investment and training, server infrastructure and maintenance, backup electrical systems due to lack of reliable electricity, and ongoing costs of feature implementation as needs evolve. Unfortunately, while many EMR vendors with a presence in LMICs are well-equipped to provide a system adequate for outpatient and basic inpatient care, most have never implemented features that are required for critical care, such as flowsheets, drip records, ventilator flowsheets, computerized order entry that can deal with complex medication scheduling, and basic order error checking, etc. In that case, these features must be implemented, which can take significant time and effort, or else parallel systems are created, which reduces efficiency.

Other less obvious challenges not commonly present in HICs blunt some of the promise of EMRs. For instance, patients may deliberately register under different names each time they are admitted, to avoid paying outstanding bills—making it so that you can no longer take advantage of historical data in the system. Poor internet access in satellite centers of care makes it difficult to implement a synchronized record. Computer literacy amongst staff, including ancillary clerical staff that must interact with the record, is not a given, which must be considered when planning initial implementation. Once implemented, record failure can be more catastrophic than a non-electronic system.

### Devices, Equipment and Supplies

Innovation in medical technology, equipment and supplies has continued to advance over the past decade. Many innovations were reviewed in our last article under “stuff,” however innovations not previously highlighted include the local production of hand cleansers (82), improvised peritoneal dialysis catheters and fluids (83), filtered sunlight phototherapy (84) and improvised electrical phototherapy machines (85).

Acute respiratory failure remains one of the leading causes of death in children under 5-years old, and therefore it should be the focus of much of the research and technological developments. Examples of on the horizon equipment advancements include the recently developed “National Hospital Abuja (NHA) Bubble CPAP Model” (86) which uses a Y-connector to blend humidified oxygen and medical air, a simple graduated container as the pressure generator, and an oxygen prong that provides the interface along with the inspiratory and expiratory limbs. An oxygen concentrator can be used with the NHA customized CPAP which decreases need for oxygen cylinders which are a valuable resource (86).

Other up and coming innovations related to respiratory support technology include, but are not limited to, the development of low-cost oxygen blenders (87, 88), humidifiers (89), and bi-level ventilators (90). Although pulse oximeters have been increasingly used and supported by companies like Lifebox^TM^ many more are needed (91) for more advanced affordable cardiac and respiratory monitoring. Most critical care units in low-resource settings are managed without the availability of blood gases or end-tidal carbon dioxide monitoring using pulse oximetry, respiratory rate, and clinical exam alone to determine how and when to change ventilator settings or wean to extubation. Untold numbers die because of limited availability of rapid accurate point-of-care testing (92).

Finally, ultrasound including point-of care ultrasound, is increasingly being used to improve the diagnosis and treatment in acute care critical illnesses and conditions like severe pneumonia and pericardial effusions (93). Unfortunately, this technology is often still outside the realm of many practioners due to both cost and availability. It is essential to continuing to focus on adaptations to these machines that will increase their accessibility in LMICs, along with training and research-based protocols so that this can become a standard part of care.

## Conclusion

Providing high quality PCC to the global community is an ethical and moral obligation. We are well-positioned to meet this challenge if we continue to work collaboratively with our partners around the world building on our past experiences and lessons learned and advancing care through research, education and training, virtual collaboration and information sharing, and ongoing technological innovations for medical devices and supplies. If the pandemic has taught us anything it has taught us that we can rise to the occasion and modify care in a way that can be expansive and lead to new frontiers in PCC.

## Author Contributions

AB and TS: conceptualization, reviewing literature, drafting, writing, critically editing for important intellectual content, final approval of the version to be published, and agree to be accountable for all aspects of the work in ensuring that questions related to the accuracy or integrity of any part of the work are appropriately investigated and resolved. LD: reviewing literature, drafting, co-writing key section critically editing for important intellectual content, final approval of the version to be published, and agree to be accountable for all aspects of the work in ensuring that questions related to the accuracy or integrity of any part of the work are appropriately investigated and resolved. LD, MY, CS, AK, and KK: reviewing literature, drafting, writing, critically editing for important intellectual content, final approval of the version to be published, and agree to be accountable for all aspects of the work in ensuring that questions related to the accuracy or integrity of any part of the work are appropriately investigated and resolved. AS and RO: reviewing literature, critically editing for important intellectual content, final approval of the version to be published, and agree to be accountable for all aspects of the work in ensuring that questions related to the accuracy or integrity of any part of the work are appropriately investigated and resolved. All authors contributed to the article and approved the submitted version.

## Conflict of Interest

The authors declare that the research was conducted in the absence of any commercial or financial relationships that could be construed as a potential conflict of interest.

## Publisher's Note

All claims expressed in this article are solely those of the authors and do not necessarily represent those of their affiliated organizations, or those of the publisher, the editors and the reviewers. Any product that may be evaluated in this article, or claim that may be made by its manufacturer, is not guaranteed or endorsed by the publisher.

## Abbreviations

ACES: academic competencies series
AAP: American academy of pediatrics
bCPAP: bubble continuous positive airway pressure
CPAP: continuous positive airway pressure
EMR: electronic medical records
TAT, emergency triage, assessment: and treatment
HBB: helping babies breathe
FDA: federal drug administration
FOAMed: free open access medical education
HICs: high income countries
LMICs: low- and middle-income countries
NHA: national hospital abuja
NIH: national institutes of health
NICST: networking for improving critical care systems and training
PALISI: pediatric acute lung injury and sepsis investigators
PCC: pediatric critical care
PICUs: pediatric intensive care units
PALS: pediatric acute life support
PAN: pediatric association of nigeria
PEARLS: pediatric educational adaptations for resource limited settings
PECC-K: pediatric emergency and critical care-kenya
SUGAR: simulation use away rotations
USAID, simulation use for global away rotations: united states agency for international development for international development
WHO: world health organization.
